# Integrated analysis of tumor differentiation genes in pancreatic adenocarcinoma

**DOI:** 10.1371/journal.pone.0193427

**Published:** 2018-03-29

**Authors:** Ting Xi, Guizhi Zhang

**Affiliations:** 1 Department of Gastroenterology, First People’s Hospital of Liaocheng, Liaocheng, Shandong Province, China; 2 Department of Gastroenterology, Second People’s Hospital of Liaocheng, Liaocheng, Shandong Province, China; University of South Alabama Mitchell Cancer Institute, UNITED STATES

## Abstract

**Background:**

Tumor differentiation is an important process in the development of cancer. It is valuable to identify key differentiation related genes in the prognosis and therapy of pancreatic adenocarcinoma.

**Methods:**

The mRNA expression data were downloaded from the Cancer Genome Atlas database. Then, differentially expressed tumor differentiation related genes were identified. Additionally, Gene Ontology functional categories and Kyoto Encyclopedia of Genes and Genomes biochemical pathway was used to explore the function. In addition, receiver operating characteristic and survival analysis were carried out to assess the diagnosis and prognosis value. Finally, the electronic validation of selected tumor differentiation related genes was performed.

**Results:**

A total of 932 genes were identified. Among which, 8 genes including *JUB*, *ERLIN1*, *HMGA2*, *FAM110B*, *EGFR*, *MCM2*, *TCTA* and *SSTR1* were differentially expressed in all different tumor differentiation grades. Functional analysis revealed those genes between highly differentiated and other differentiation were remarkably enriched in pancreatic adenocarcinoma and cell cycle pathway. Finally, *ERLIN1*, *HMGA2*, *FAM110B*, *EGFR*, *MCM2*, *BCL2L1*, *E2F1* and *RAC1* were associated with the survival time of pancreatic adenocarcinoma patient. Among these genes, *JUB*, *ERLIN1*, *FAM110B*, *MCM2* and *BCL2L1* also had a diagnosis value for pancreatic adenocarcinoma. Additionally, the expression trend of *JUB*, *HMGA2* and *MCM2* was increased along with the tumor differentiation grades. And the expression trend of *FAM110B* was decreased along with the tumor differentiation grades. The electronic validation result was consistent with the bioinformatics analysis.

**Conclusions:**

12 tumor differentiation related genes including *JUB*, *ERLIN1*, *HMGA2*, *FAM110B*, *EGFR*, *MCM2*, *TCTA*, *SSTR1*, *BCL2L1*, *E2F1*, *RAC1* and *STAT1* played crucial roles in the differentiation of pancreatic adenocarcinoma.

## Introduction

Pancreatic adenocarcinoma is an important leading cause of cancer-related mortality in the Western world [1]. Generally, the vast majority of pancreatic adenocarcinoma patients present with no specific symptoms until appear jaundice and weight loss, signs of advanced stage of pancreatic adenocarcinoma [2]. The lethal characteristic of pancreatic adenocarcinoma is the high metastatic potential to distant organs and the lymphatic system [3]. Additionally, lack of effective chemotherapy also contributes to the high mortality of pancreatic adenocarcinoma patients [4].

UP to now, the therapy of pancreatic adenocarcinoma remains a great challenge in clinical oncology on account of the similar rate between incidence and mortality [5]. According to the report of cancer statistics in 2015, in contrast to the steady increase in survival for most cancers, advance has been slow for pancreatic adenocarcinoma, for which the 5-year relative survival is recent 7% [6]. Therefore, advances in understanding the molecular biology and discovery of novel therapeutic targets will contribute to clinical management of pancreatic adenocarcinoma.

Based on the degree of tumor differentiation, pancreatic adenocarcinoma is divided into four grades including highly differentiated (G1), moderately differentiated (G2), poorly differentiated (G3) and un-differentiation (G4). Moreover, patients with poorly differentiated tumors have a worse prognosis than those with well differentiated tumors [7]. It is noted that The Cancer Genome Atlas (TCGA, https://tcga-data.nci.nih.gov/tcga/) database is a publicly funded project that is consisted of multidimensional data of different cancers in patients at DNA, RNA and protein levels. Therefore, the objective of our study is to identify the tumor differentiation related genes based on the TCGA dataset, shedding light on molecule mechanism and identifying new therapy targets for the treatment of pancreatic adenocarcinoma.

## Material and methods

### The Cancer Genome Atlas dataset

The Cancer Genome Atlas (TCGA, https://tcga-data.nci.nih.gov/tcga/) is a publicly funded project that is consisted of multidimensional data of different cancers in patients at DNA, RNA and protein levels. In this study, TCGA dataset was used to retrieve the gene expression data in pancreatic adenocarcinoma.

### Identification of tumor differentiation related differentially expressed genes

In order to find potential genes in the process of tumor differentiation, differentiation related transcription sequencing data were analyzed by linear by linear association test in 176 pancreatic adenocarcinoma patients. The lbl.Test function analysis in the cion package of R software was used to identify differentiation related genes. The detailed process was as follows: Firstly, the sample was divided into four express quantity interval according to the quartiles of each gene expression quantity. Secondly, the correlation between express quantity interval and tumor grade was tested. Thirdly, tumor differentiation related genes were selected according to the threshold of p < 0.01. Finally, the expression of each gene in different tumor differentiation grades was further analyzed through tukey's honest, and p < 0.05 was considered the statistical significant difference.

### Functional annotation of tumor differentiation related differentially expressed genes

The Gene Ontology (GO) function was used to enrich the biological function of genes in biological progress, cellular component and molecular function [8]. The Kyoto Encyclopedia of Genes and Genomes (KEGG) dataset is applied to analyze gene function and genome information systematically, which is helpful in studying gene expression in a network. It is consisted of six subdata including PATHWAY, BRITE, MODULE, DISEASE, GENES and GENOME. Among which, PATHWAY dataset contains the most advanced functional information such as metabolism, membrane transport, signal transmission and cell cycle. To further understand the biological function of tumor differentiation related genes in pancreatic adenocarcinoma, we performed the GO functional categories and the KEGG biochemical pathway using GeneCodis3 (http://genecodis.cnb.csic.es/analysis). False discovery rate < 0.05 was considered to be statistically significant.

### Receiver operating characteristic (ROC) analyses

In order to assess the diagnostic value of tumor differentiation related differentially expressed genes in pancreatic adenocarcinoma, we performed the receiver operating characteristic (ROC) analyses through the pROC package in R language. The area under the curve (AUC) under binomial exact confidence interval was calculated and the ROC curve was generated.

### Survival analysis of tumor differentiation related differentially expressed genes

The R package (3.4.0 version, 2017) was used to explore the prognostic ability to predict patient survivability of identified tumor differentiation related differentially expressed genes in the TCGA database.

### Electronic validation of tumor differentiation related genes in GEO database

We employed the Gene Expression Omnibus (GEO) database to validate the expression of selected tumor differentiation related genes. We compared the expression levels of these genes in different tumor differentiation grades including G1, G2, G3 and G4. The difference of expression levels was displayed by box-plots.

## Results

### Clinical information

In this study, the mRNA expression data of patients with pancreatic adenocarcinoma were downloaded from the platform of UNC_IlluminaHiSeq_RNASeqV2 in TCGA data portal. Downloaded mRNA expression data were standardized. Those patients who had a tumor grade of GX were ruled out for this study. Finally, 176 patients with clinical tumor grade information and mRNA expression data were included in this study. Among which, 31 were highly differentiated (G1), 95 were moderately differentiated (G2), 48 were poorly differentiated (G3) and 2 were un-differentiated (G4). G3 and G4 were combined together (G3/G4). Detailed information of pancreatic adenocarcinoma patients was presented in Table 1.

**Table 1:** Information of patients with pancreatic adenocarcinoma.

Parameter		Patients (N = 176)
Gender	Male	97
	Female	79
Age	>60 (Mean ± SD)	70.72±6.43
	≤60 (Mean ± SD)	51.97±6.08
Race	White	156
	Black or African American	6
	Asian	10
Vital status	Unknown	3
	Alive	117
	Dead	59
Follow-up(days)	>5 years (Mean ± SD)	5.73±0.68
	<5 years(Mean ± SD)	0.90±0.89
Tumor differentiation	G1	31
	G2	95
	G3/G4	50
Radiation therapy	Yes	31
	No	102
	Unknown	42
	Not Available	1
	Yes	1
History of neoadjuvant treatment	No	175
	Yes	89
	No	30
Targeted molecular therapy	Not applicable	57

G1: highly tumor differentiated; G2: moderately tumor differentiated; G3/G4: poorly tumor differentiation and un-differentiated.

### Tumor differentiation related differentially expressed genes

**Fig 1:**
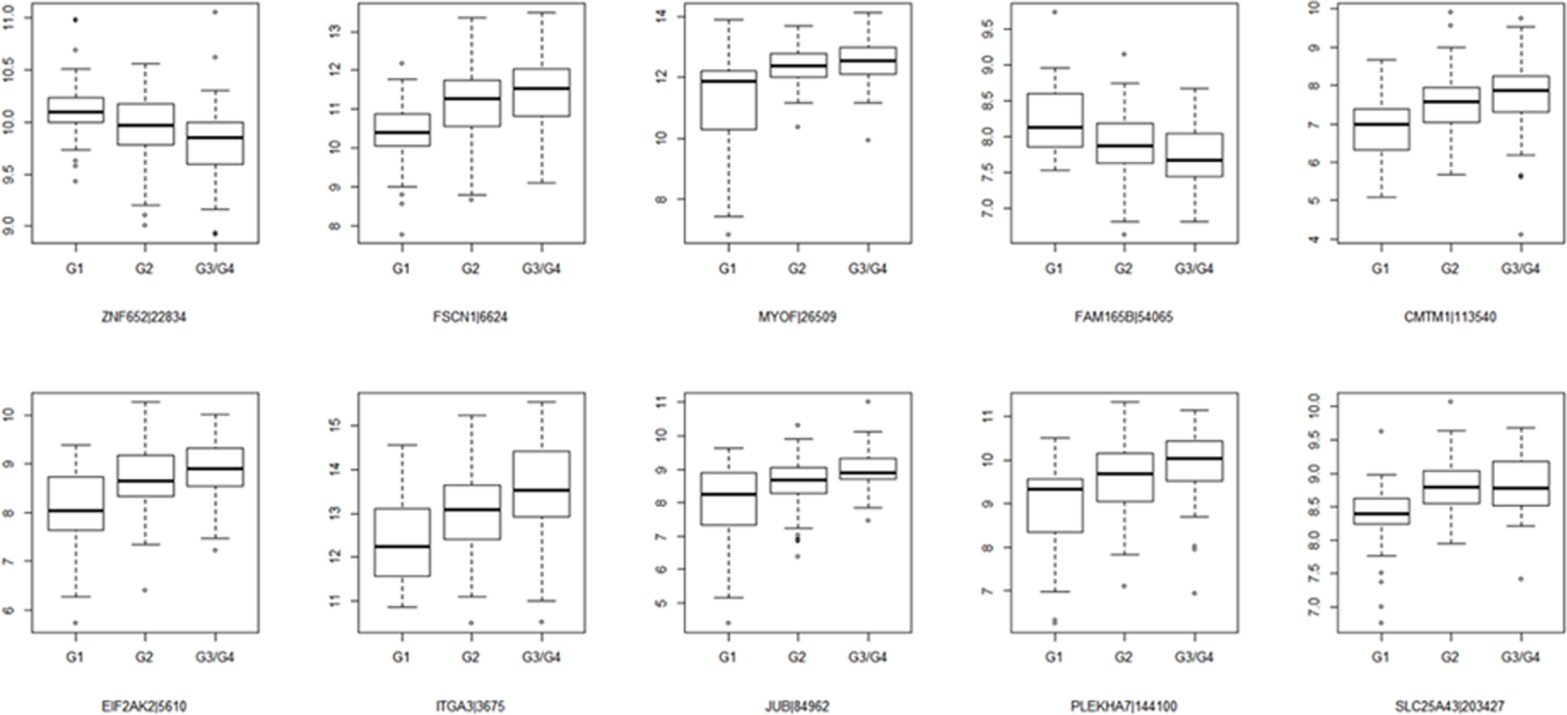
The Box-plot of top 10 tumor differentiation related genes in pancreatic adenocarcinoma. The x-axis shows the tumor differentiation grades and y-axis shows expression reads counts.

**Fig 2:**
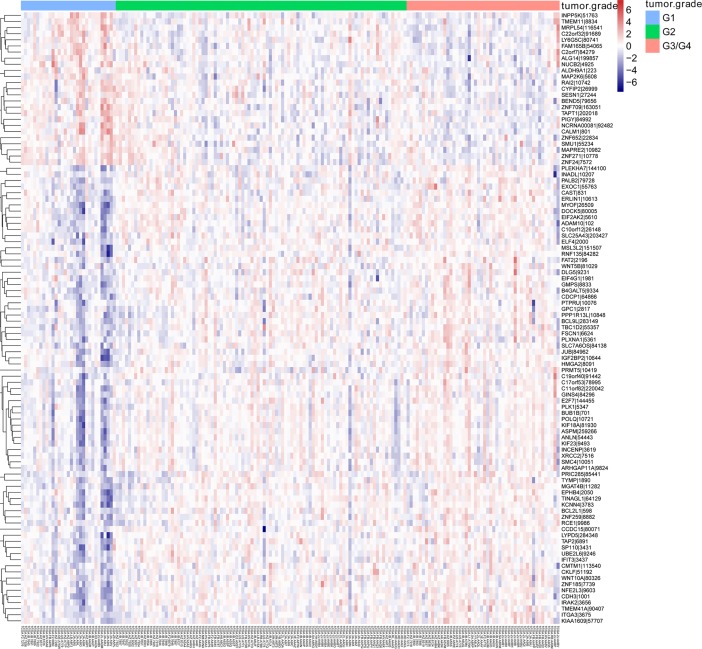
The heat map of top 100 tumor differentiation related genes in pancreatic adenocarcinoma. Diagram presents the result of a two-way hierarchical clustering of top 100 tumor differentiation-related genes and samples. The clustering is constructed using the complete-linkage method together with the Euclidean distance. Each row represents a gene and each column, a sample. The gene clustering tree is shown on the right. The colour scale illustrates the relative level of gene expression: red, below the reference channel; blue, higher than the reference. G1: highly tumor differentiated; G2: moderately tumor differentiated; G3/G4: poorly tumor differentiation and un-differentiated.

**Fig 3:**
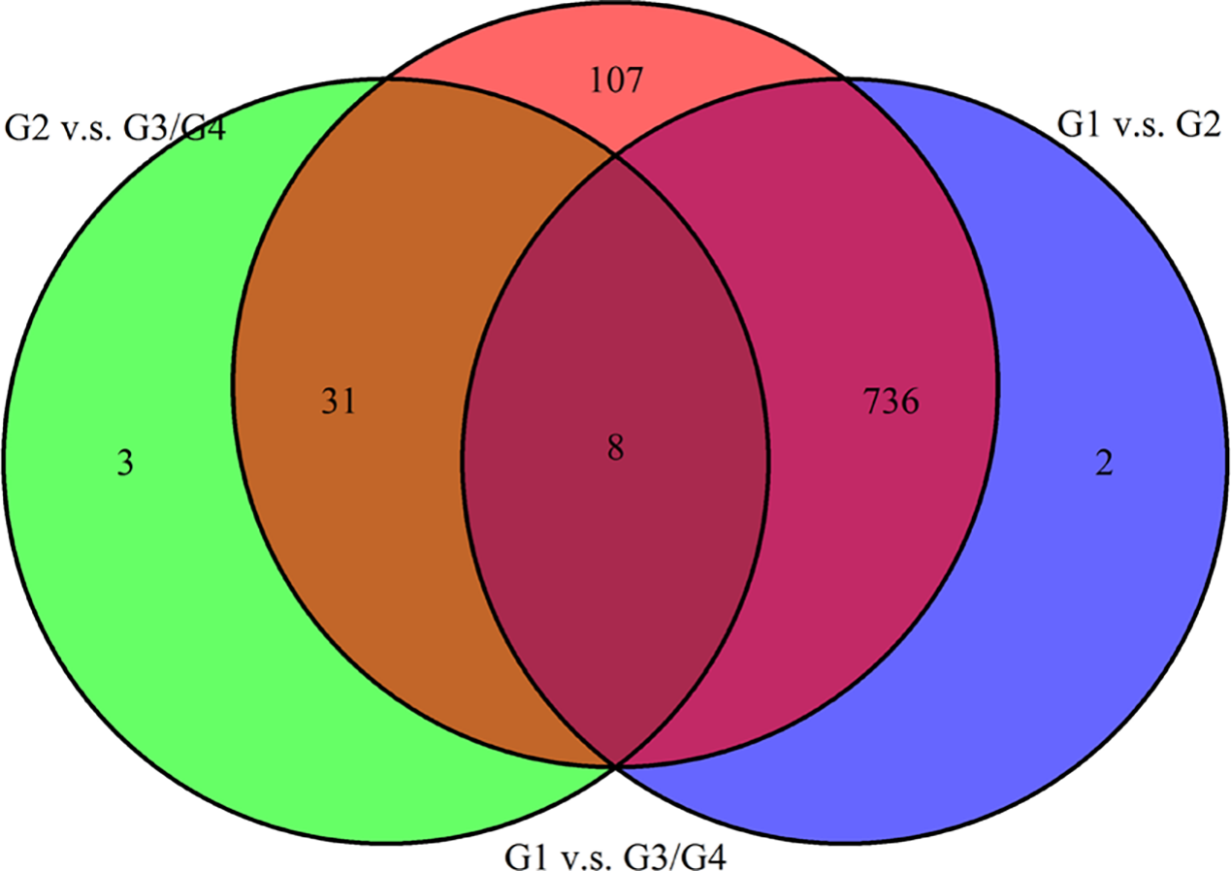
The Venn of genes expression overlaps in different tumor differentiation grades in pancreatic adenocarcinoma. G1: highly tumor differentiated; G2: moderately tumor differentiated; G3/G4: poorly tumor differentiation and un-differentiated.

Based on transcriptome sequencing data analysis in the context of tumor differentiation grade, 932 (614 positively associated and 318 negatively associated) related genes were identified in pancreatic adenocarcinoma. The top 10 tumor differentiation related genes were shown in Table 2. The Box-plot of top 10 tumor differentiation related genes was also shown in Fig 1. In addition, the heat map of top 100 tumor differentiation related genes was presented in Fig 2. Additionally, tukey's honest significant difference was used to further investigate the different expression of each gene in different tumor differentiation grades. The analysis results revealed that there were 746 differentially expressed genes between G1 and G2, 882 differentially expressed genes between G1 and G3/G4 and 42 differentially expressed genes between G2 and G3/G4. The Venn of genes expression overlaps in diverse tumor differentiation grades was shown in Fig 3. It is worth mentioning that 8 genes including *JUB*, *ERLIN1*, *HMGA2*, *FAM110B*, *EGFR*, *MCM2*, *TCTA* and *SSTR1* were differentially expressed in distinct tumor differentiation grades.

**Table 2:** The top 10 tumor differentiation-related genes.

Gene ID	Gene symbol	Mean G1	Mean G2	Mean G3/G4	P Value	Association
22834	*ZNF652*	10.1460	9.9536	9.8123	7.94E-06	Negative
6624	*FSCN1*	10.3484	11.1331	11.4693	1.26E-05	Positive
26509	*MYOF*	11.0974	12.3869	12.5488	1.26E-05	Positive
54065	*FAM165B*	8.2168	7.8843	7.7171	1.26E-05	Negative
113540	*CMTM1*	6.9000	7.5645	7.7247	1.99E-05	Positive
5610	*EIF2AK2*	8.0822	8.7083	8.8838	1.99E-05	Positive
3675	*ITGA3*	12.3691	13.0239	13.4195	1.99E-05	Positive
84962	*JUB*	7.9763	8.6089	8.9946	1.99E-05	Positive
144100	*PLEKHA7*	8.9381	9.6629	9.9410	1.99E-05	Positive
203427	*SLC25A43*	8.3147	8.8164	8.8370	1.99E-05	Positive

### Biological function of tumor differentiation related differentially expressed genes

**Fig 4:**
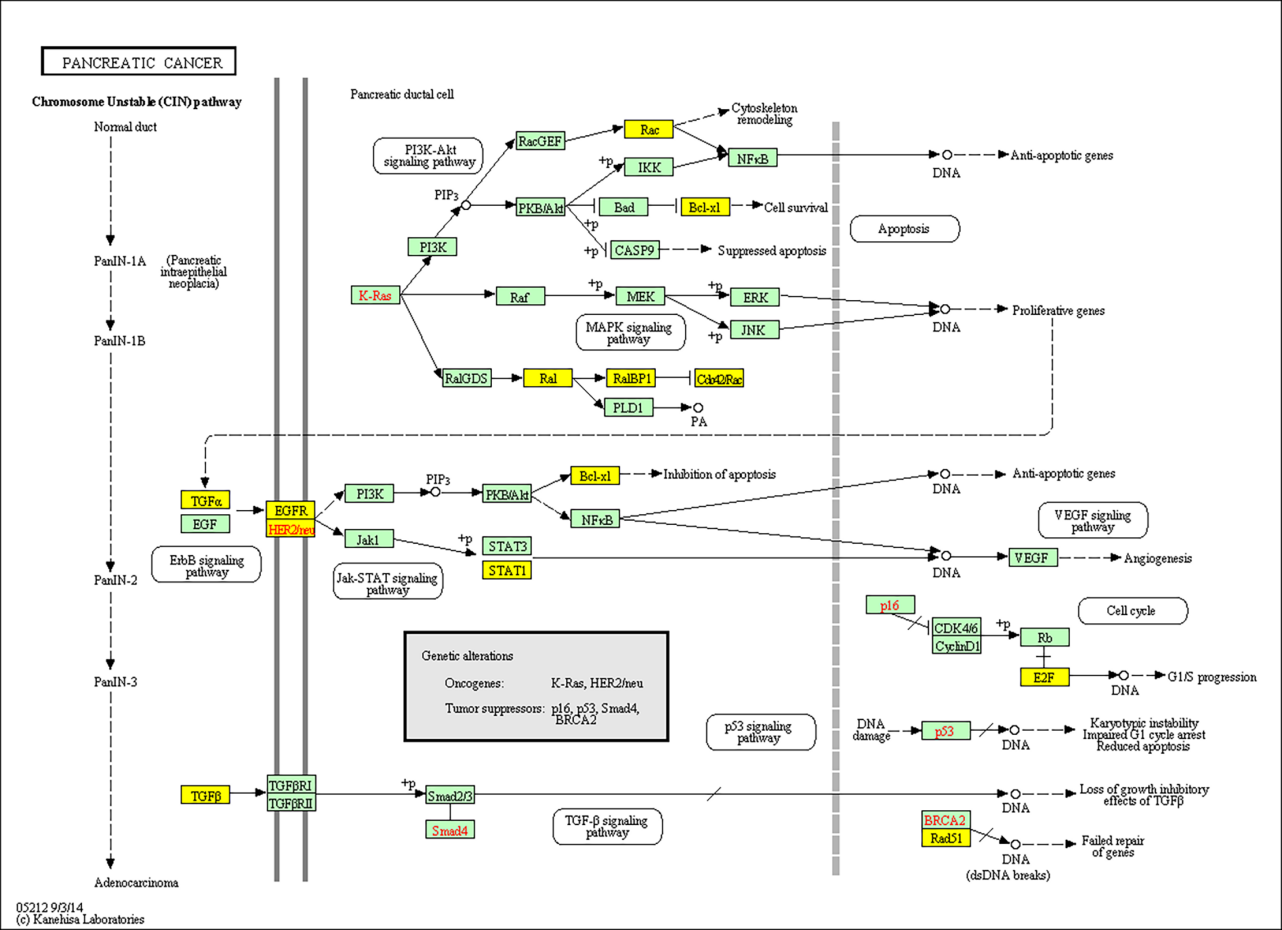
The tumor differentiation related gene enriched KEGG map of pancreatic adenocarcinoma. The yellow colours represents the enriched genes in the pathway of pancreatic adenocarcinoma.

To investigate the gene function, GO and KEGG were applied to perform function annotation in 744 differentially expressed genes (the intersection genes between G1 vs G2 and G1 vs G3/G4) and 705 genes were recognized. GO enrichment analysis revealed that these genes were significantly involved in cell division (false discovery rate = 2.15E–17), cell cycle (false discovery rate = 2.19E–17) and cell proliferation (false discovery rate = 1.17E–07). Additionally, KEGG pathway enrichment analysis showed that these genes were remarkably enriched in pathways in cancer (false discovery rate = 8.56E–07), cell cycle (false discovery rate = 3.48E–06) and pancreatic adenocarcinoma (false discovery rate = 1.10E–05). Top 15 Go terms and top 3 KEGG pathways for differentially expressed genes were listed in Table 3 and Table 4, respectively. Remarkably, 11 genes including *STAT1*, *BCL2L1*, *TGFA*, *ERBB2*, *E2F1*, *RAD51*, *RALB*, *RALBP1*, *TGFB2*, *EGFR* and *RAC1* were involved in the pathway of pancreatic adenocarcinoma. 15 genes including *MCM4*, *CCNB2*, *CDC20*, *CCNA2*, *CCND2*, *E2F1*, *CCNB1*, *TFDP2*, *TGFB2*, *PLK1*, *BUB1B*, *PKMYT1*, *TTK*, *BUB1* and *MCM2* were involved in the cell cycle. The KEGG map of pancreatic adenocarcinoma was showed in Fig 4.

**Table 3:** Top 15 Go terms for differentially expressed genes in pancreatic adenocarcinoma.

GO ID	GO Term	Count	False discovery rate
**Biological process**			
GO:0051301	cell division (BP)	39	2.15E-17
GO:0007049	cell cycle (BP)	47	2.19E-17
GO:0000278	mitotic cell cycle (BP)	35	1.56E-13
GO:0000087	M phase of mitotic cell cycle (BP)	21	2.11E-13
GO:0007067	mitosis (BP)	26	7.60E-12
GO:0000236	mitotic prometaphase (BP)	18	3.95E-11
GO:0001525	angiogenesis (BP)	21	5.42E-09
GO:0008283	cell proliferation (BP)	27	1.17E-07
GO:0007165	signal transduction (BP)	55	4.06E-06
GO:0006468	protein phosphorylation (BP)	28	4.39E-06
GO:0006915	apoptotic process (BP)	35	6.57E-06
GO:0031581	hemidesmosome assembly (BP)	6	1.02E-05
GO:0006355	regulation of transcription, DNA-dependent (BP)	66	1.42E-05
GO:0045892	negative regulation of transcription, DNA-dependent (BP)	27	1.47E-05
GO:0006260	DNA replication (BP)	16	1.50E-05
**Molecular function**			
GO:0005515	protein binding (MF)	230	2.76E-39
GO:0005524	ATP binding (MF)	97	2.78E-21
GO:0000166	nucleotide binding (MF)	116	8.85E-20
GO:0046872	metal ion binding (MF)	108	1.38E-07
GO:0003677	DNA binding (MF)	76	2.57E-07
GO:0016787	hydrolase activity (MF)	49	1.16E-06
GO:0042803	protein homodimerization activity (MF)	32	3.63E-06
GO:0008270	zinc ion binding (MF)	76	5.26E-06
GO:0019901	protein kinase binding (MF)	20	8.90E-06
GO:0004674	protein serine/threonine kinase activity (MF)	24	8.46E-05
GO:0042802	identical protein binding (MF)	20	2.76E-04
GO:0008134	transcription factor binding (MF)	18	4.85E-04
GO:0005509	calcium ion binding (MF)	32	5.00E-04
GO:0005488	binding (MF)	34	5.10E-04
GO:0004386	helicase activity (MF)	12	5.47E-04

**Table 4:** Top 3 KEGG pathways for differentially expressed genes in pancreatic adenocarcinoma.

KEGG ID	KEGG term	Count	False discovery rate	Genes
hsa05200	Pathways in cancer	26	8.56E-07	WNT10A, MET, STAT1, LAMA3, BCL2L1, CTNNA1, FZD6, TGFA, ERBB2, ITGA3, CASP8, E2F1, RAD51, RALB, PIAS3, FADD, RALBP1, LAMB3, TGFB2, PML, EGFR, CKS1B, LAMC2, RAC1, SLC2A1, CTNNB1
hsa04110	Cell cycle	15	3.48E-06	MCM4, CCNB2, CDC20, CCNA2, CCND2, E2F1, CCNB1, TFDP2, TGFB2, PLK1, BUB1B, PKMYT1, TTK, BUB1, MCM2
hsa05212	Pancreatic cancer	11	1.10E-05	STAT1, BCL2L1, TGFA, ERBB2, E2F1, RAD51, RALB, RALBP1, TGFB2, EGFR, RAC1

### ROC curve analysis

**Fig 5:**
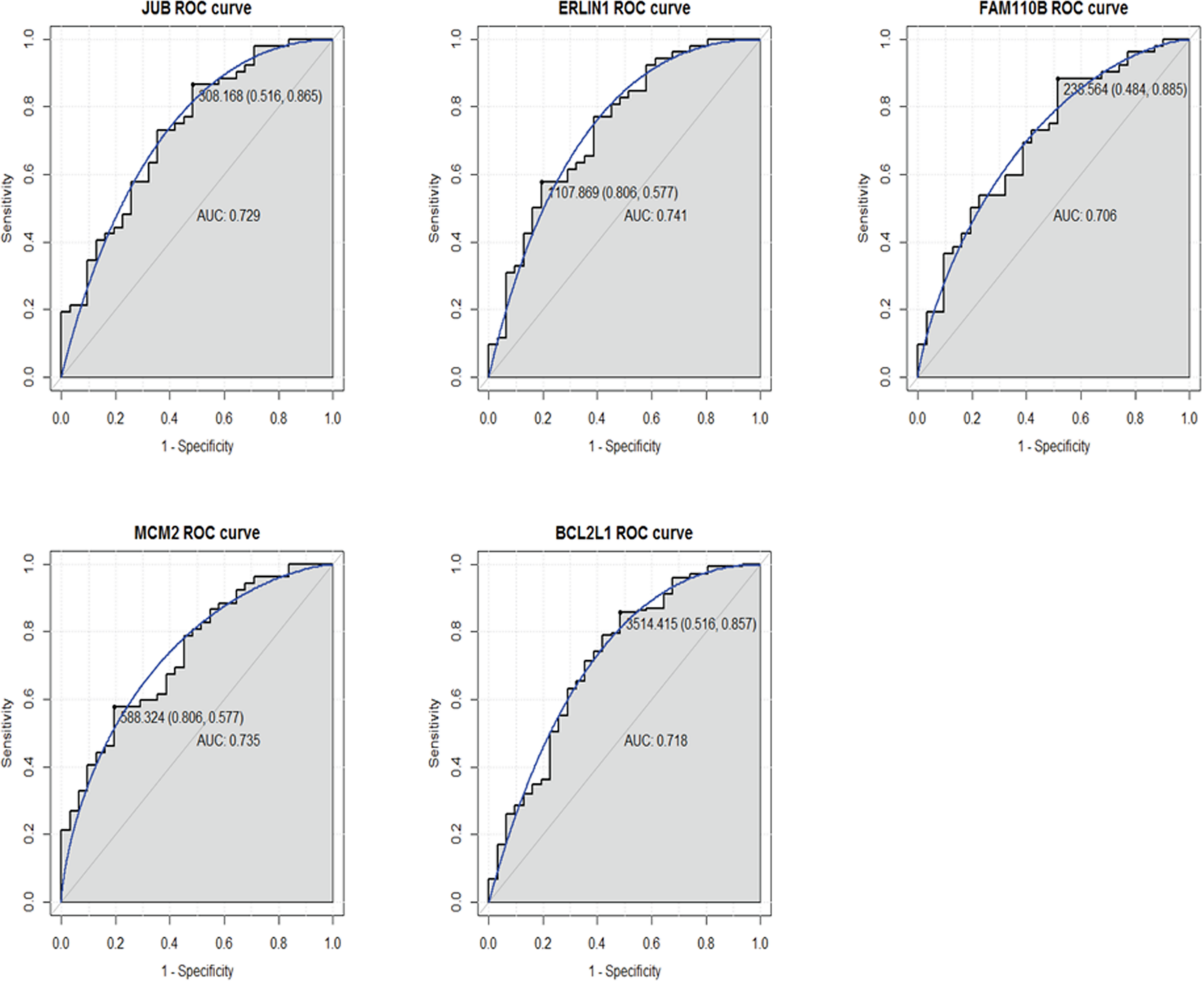
ROC curves of selected differentially expressed genes between pancreatic adenocarcinoma and healthy controls. The ROC curves were used to show the diagnostic ability of these selected differentially expressed genes with 1-specificity (the proportion of false positive) and sensitivity (the proportion of true positive). The x-axis shows 1-specificity and y-axis shows sensitivity.

We performed ROC curve analyses and calculated the AUC to assess the discriminatory ability of five tumor differentiation related differentially expressed genes in the TCGA dataset. The AUC of these genes including *JUB* (0.729), *ERLIN1* (0.741), *FAM110B* (0.706), *MCM2* (0.735) and *BCL2L1* (0.718) was more than 0.7 (Fig 5). For pancreatic adenocarcinoma diagnosis, the 1-specificity (proportion of false positive) and sensitivity (proportion of true positive) of *JUB* was 51.6% and 86.5%, respectively; the 1- specificity and sensitivity and of *ERLIN1* was 80.6% and 57.7%, respectively; the 1- specificity and sensitivity and of *FAM110B* was 48.4% and 88.5%, respectively; the 1- specificity and sensitivity and of *MCM2* was 80.6% and 57.7%, respectively; the 1- specificity and sensitivity and of *BCL2L1* was 51.6% and 85.7%, respectively.

### Survival prediction of tumor differentiation related differentially expressed genes

**Fig 6:**
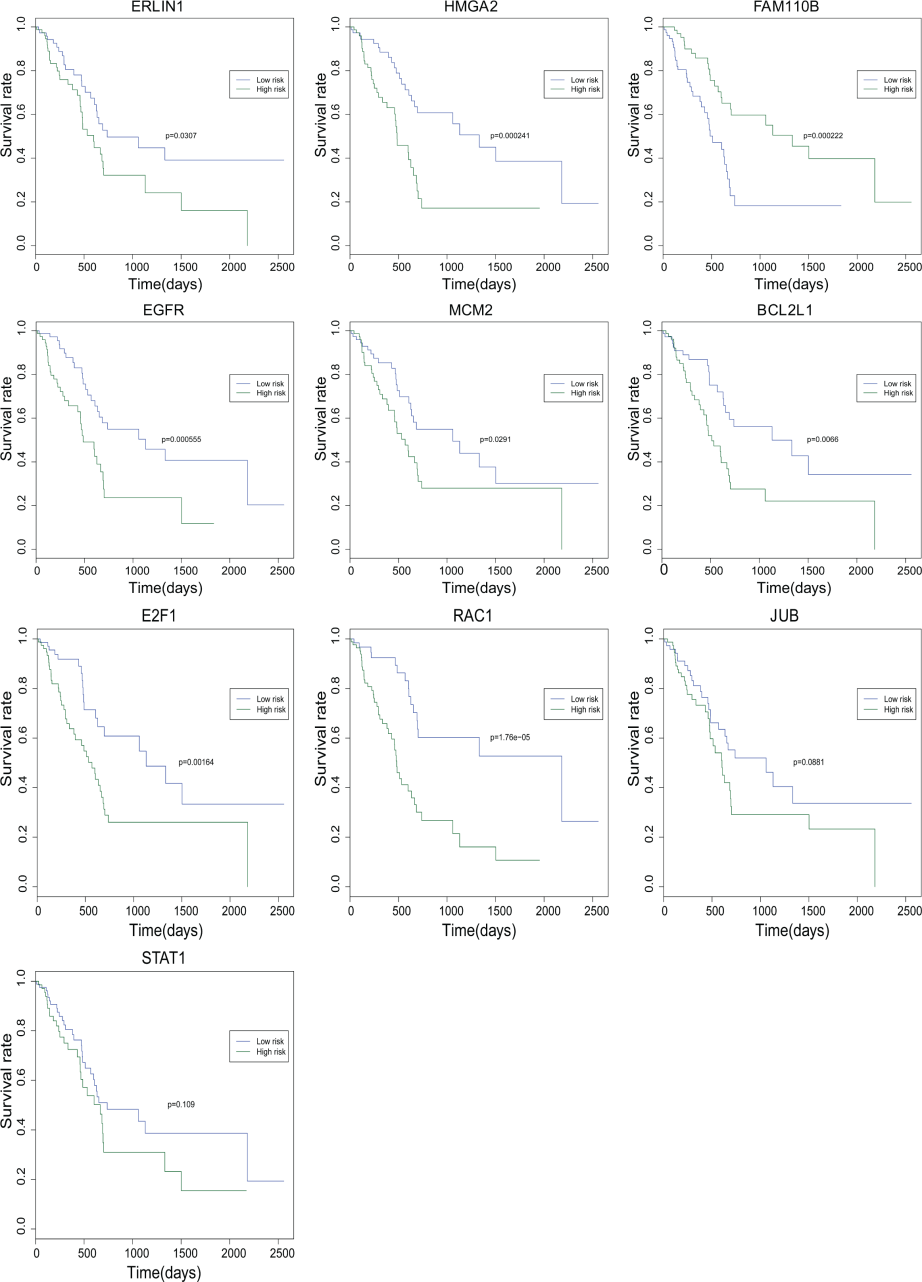
The survival curves of ten tumor differentiation-related genes in pancreatic adenocarcinoma. The x-axis shows the survive time (days) and y-axis shows survival rate.

To analyze the potential prognostic characteristics of tumor differentiation related genes in pancreatic adenocarcinoma, 6 differentially expressed genes in all different tumor differentiation grades (*JUB*, *ERLIN1*, *HMGA2*, *FAM110B*, *EGFR* and *MCM2*) and four differentially expressed genes that enriched in pancreatic adenocarcinoma signaling pathway (*BCL2L1*, *E2F1*, *RAC1* and *STAT1*) were analyzed using the R package (3.4.0 version, 2017). Finally, 8 differentially expressed genes (*ERLIN1*, *HMGA2*, *FAM110B*, *EGFR*, *MCM2*, *BCL2L1*, *E2F1* and *RAC1*) were considered to be significantly negatively associated with survival (P < 0.05) time of pancreatic adenocarcinoma patients. However, *JUB* and *STAT1* were not remarkably related to the survival time of pancreatic adenocarcinoma patients. The survival curves of above 10 genes were illustrated in Fig 6.

### Electronic validation of tumor differentiation related genes in GEO database

**Fig 7:**
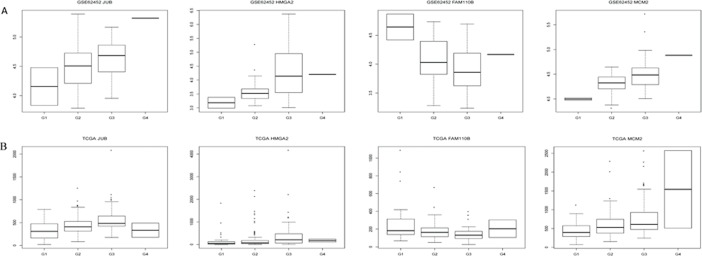
The validation of the expression levels of JUB, HMGA2, FAM110B and MCM2 in pancreatic adenocarcinoma based on GEO database. The x-axis shows the case and normal groups and y-axis shows expression reads counts. (A) The expression validation in the GEO database (B): The expression in the TCGA sequencing data.

Based on the sequencing data, four tumor differentiation related genes (*JUB*, *HMGA2*, *FAM110B* and *MCM2*) were selected to perform the expression validation by GEO database (Fig 7A). Different expression levels of these genes in G1, G2, G3 and G4 were analyzed and depicted through box-plots. The box-plots were displayed by median and inter-quartile range visually. The expression levels of *JUB*, *HMGA2* and *MCM2* were increased gradually along with the tumor differentiation grades. However, the expression level of *FAM110B* was decreased gradually along with the tumor differentiation grades. The expression trend of these genes in different tumor differentiation grades was consistent with the TCGA sequencing data (Fig 7B).

## Discussion

Pancreatic adenocarcinoma is the fourth most common cause of cancer-related mortality [9]. It is worth mentioning that tumor differentiation is a universal process in the development of different cancers. Therefore, the objective of this study was to identify tumor differentiation related genes in pancreatic adenocarcinoma. In this study, we found 932 tumor differentiation related genes. After different expression analysis of each gene in diverse tumor differentiation grades, 8 genes were differentially expressed in all grades. GO and KEGG annotation in 744 differentially expressed genes (the intersection genes between G1 vs G2 and G1 vs G3/G4) revealed that pancreatic adenocarcinoma and cell cycle were significantly enriched pathways. ROC and survival prediction analysis identified several genes that had a diagnosis and prognosis value for pancreatic adenocarcinoma. In conclusion, 8 tumor differentiation related genes (*JUB*, *ERLIN1*, *HMGA2*, *FAM110B*, *EGFR*, *MCM2*, *TCTA* and *SSTR1*), pancreatic adenocarcinoma pathway related genes (*BCL2L1*, *E2F1*, *RAC1* and *STAT1*) and cell cycle pathway related genes (*BUB1*, *BUB1B*, *CCNA2*, *CCNB2*, *CCND2*, *CDC20*, *PLK1*, *TGFB2* and *TTK*) played fatal roles in the development of pancreatic adenocarcinoma.

Ajuba LIM protein (JUB, also called AJUBA) is a well-known cancer associated protein, which is involved in tumor invasion and migration [10]. It has been reported that the inactivation of JUB is implicated in deregulation of cell differentiation in head and neck squamous cell carcinomas [11]. Significantly, we found that *JUB* was differentially expressed in all tumor differentiation grades and the expression level increased gradually along with the tumor differentiation grades, which suggested the positive association with tumor differentiation of pancreatic adenocarcinoma. Furthermore, ROC analysis indicated that *JUB* had a diagnostic value for pancreatic adenocarcinoma patients.

ER lipid raft associated 1 (*ERLIN1*) encodes members of the prohibitin that defines lipid-raft-like domains of the endoplasmic reticulum [12]. It is reported that the expression of ERLIN1 is decreased in laser capture microdissection gastric carcinomas compared to histologic macrodissection [13]. Herein, we first found that the expression of *ERLIN1* was positively related to tumor differentiation of pancreatic adenocarcinoma. In addition, *ERLIN1* had a diagnosis value and associated with the survival time of pancreatic adenocarcinoma patients. Therefore, we concluded that *ERLIN1* played crucial roles in tumor differentiation and could be a diagnosis and prognosis marker for pancreatic adenocarcinoma.

High mobility group AT-hook 2 (HMGA2) is a transcription factor primarily expressed in the mesenchyme and regulates mesenchymal proliferation and differentiation [14, 15]. It is demonstrated that immunoreactivity of HMGA2 is correlated to poor differentiation in pancreatic ductal adenocarcinoma [16]. Our study first found the different expression of *HMGA2* in tumor differentiation of pancreatic adenocarcinoma. The expression of *HMGA2* was increased along with the tumor differentiation grades. In addition, we also found that *HMGA2* was significantly related to the survivability of pancreatic adenocarcinoma. Thus, we supposed that *HMGA2* was a promising molecular target for pancreatic adenocarcinoma therapy and prognosis.

Cell mitosis is an important process in the cell cycle, which contributes to the biological development of cell differentiation. Family with sequence similarity 110 member B (FAM110B, also called C8orf72) accumulates at the spindle poles and centrosomes in mitosis and aberrant expression will influence cell cycle progression in G1 phase [17]. It is stated that FAM110B is a survival predictor of breast cancer stem cells [18]. Herein, we found that the expression of *FAM110B* was decreased along with the tumor differentiation grades, which showed that *FAM110B* was negatively associated with tumor differentiation and could be served as diagnosis and survival marker of pancreatic adenocarcinoma.

It is suggested that the activation of epidermal growth factor receptor (EGFR) affects cellular growth, proliferation and differentiation [19]. By contrast, the blockade of EGFR expression reduces the growth and metastatic potential of pancreatic tumor [20]. It is suggested that the major role of EGFR in pancreatic tumorigenesis is controlling the differentiation of neoplastic precursors [21]. Additionally, high expression of EGFR has been related to shorter survival of pancreatic adenocarcinoma [22]. Herein, we found that the expression of *EGFR* was associated with tumor differentiation and survivability of pancreatic adenocarcinoma, which was in line with previous reports.

Some studies have demonstrated that the minichromosome maintenance complex component 2 (MCM2) is not only the marker of cellular proliferation also required for cell cycle [23]. Moreover, it is reported that withdrawal of cells from the cell cycle into the differentiated state is accompanied by down-regulated expression of MCM2 [24, 25]. It is worth mentioning that it is up-regulation in primary pancreatic tumors compared to normal pancreas [26]. In this study, we found that *MCM2* was increased along with the tumor differentiation grades, which further demonstrated the crucial role of *MCM2* in cell differentiation of pancreatic adenocarcinoma. Significantly, we also found the diagnosis and prognosis value of *MCM2* in pancreatic adenocarcinoma.

Generally, T-cell leukemia translocation altered (*TCTA*) mRNA is expressed in normal tissues, with high-level of expression in the kidney [27]. Of note, the expression of TCTA is reduced in three of four small cell lung cancer cell lines [28]. Meaningfully, we first found expression of *TCTA* in different tumor differentiation grades of pancreatic adenocarcinoma, and the expression was negatively related to tumor differentiation. Therefore, our data indicated that *TCTA* could be considered as the therapy target of pancreatic adenocarcinoma.

Somatostatin (SST), a small cyclic neurpeptide, has been applied for treating pancreatic adenocarcinoma in pre-clinical trials as adjuvants on account of their inhibitory effects on cell proliferation and growth hormone release [29, 30]. It is reported that gene transfer using somatostatin receptor 1 (SSTR1) inhibits the growth of pancreatic adenocarcinoma through cell cycle arrest *in vivo* and *in vitro* [2]. Our study found that *SSTR1* was expressed in all grades of tumor differentiation of pancreatic adenocarcinoma, which further demonstrated the crucial role of *SSTR1* in the development of pancreatic adenocarcinoma.

According to the KEGG pathway analysis, we found that several tumor differentiation related genes (such as *BCL2L1*, *E2F1*, *RAC1* and *STAT1*) were remarkably enriched in pancreatic adenocarcinoma signaling pathway. Bcl-xL, BCL2 like 1 (*BCL2L1*) encoded protein expressed in various malignant tumors and involved in facilitating resistance to chemotherapy [31–33]. Additionally, it is over-expression in pancreatic tumor cells [34, 35]. It is suggested that the E2F transcription factor 1 (E2F1) is both an oncogenic inducer and a tumor suppressor [36, 37]. Moreover, a direct correlation between E2F1 and cell proliferation, as well as an inverse association between E2F1 and disease-associated survival has been observed in pancreatic adenocarcinoma [38]. In this study, we found the role of *BCL2L1* and *E2F1* in the process of pancreatic adenocarcinoma, which was in line with previous researches. Significantly, we also found that *BCL2L1* had a diagnosis value for pancreatic adenocarcinoma.

Several studies report that ras-related C3 botulinum toxin substrate (RAC1) is involved in controlling cell cycle, growth and survival [39, 40]. It is worth mentioning that increased expression of RAC1 has been found in pancreatic adenocarcinoma and is correlated with patient prognosis [41, 42]. It is noted that signal transducer activator of transcription 1 (STAT1) is expressed in 88% of pancreatic adenocarcinoma tissue specimens, and the expression is inversely related to tumor differentiation of pancreatic adenocarcinoma [43]. Additionally, patients with high STAT1 have a better prognosis compared to those with low expression [44]. Herein, we found that *BCL2L1*, *E2F1*, *RAC1* and *STAT1* were expressed in pancreatic adenocarcinoma, which was consistent with previous reports. Significantly, we also found the roles of *BCL2L1*, *E2F1* and *RAC1* in tumor differentiation and survival prediction of pancreatic adenocarcinoma.

Alteration in cell cycle regulatory mechanisms plays a vital role in the tumor development. Besides pancreatic cancer, cell cycle was the common biological function of differentially expressed genes in GO and KEGG, which involved several genes including *BUB1*, *BUB1B*, *CCNA2*, *CCNB2*, *CCND2*, *CDC20*, *PLK1*, *TGFB2* and *TTK*. It is found that *BUB1* is a remarkably altered gene in CD4^+^ peripheral blood cells of pancreatic adenocarcinoma patients compared with healthy volunteers [45]. BUB1B is associated with DNA repair and known to drive the development of cancer [46, 47]. It is reported that deleterious variant in *BUB1B* gene is more frequent in patients with familial pancreatic adenocarcinoma [48]. CCNA2 has been demonstrated up-regulated in pancreatic adenocarcinoma tissue samples and significantly involved in the cell cycle pathway [49]. Nakamura T and Sato N et al found that CCNB2 was also up-regulated in human pancreatic adenocarcinoma [50, 51]. CCND2 plays an important role in the proliferation of pancreatic islet b-cell and the mRNA expression of CCND2 is rarely detected in pancreatic adenocarcinoma cell lines [52–54]. It has been found that *CCND2* is hypermethylated in the progression of pancreatic adenocarcinoma [54]. It is reported that CDC20 is up-regulated in pancreatic adenocarcinoma cells [55]. Deregulation of PLK1 occurred early in carcinogenesis and over-expression in pancreatic intraepithelial neoplasia III lesions of pancreatic adenocarcinoma patients [56]. It is noted that PLK1 pathway is a potential therapeutic target of pancreatic adenocarcinoma [57]. TGFB2 is found to be over-expressed in pancreatic adenocarcinoma [58, 59]. TTK is also over-expressed in pancreatic adenocarcinoma and plays a crucial role in maintaining the viability and proliferative potential of pancreatic adenocarcinoma cells [60]. Thus it can be seen that these genes were associated with the development of pancreatic adenocarcinoma and may play an important role in the cell cycle of pancreatic adenocarcinoma differentiation.

## Conclusions

Our study provided the molecular clues in understanding the pathological mechanism of pancreatic adenocarcinoma, especially in tumor differentiation of pancreatic adenocarcinoma. Additionally, we identified several diagnosis and survivability-related differentially expressed genes, which may be regarded as diagnosis and prognosis markers in the development of pancreatic adenocarcinoma. Of course, there were limitations to our study. Firstly, we aimed to study the biological function of differentially expressed genes between highly differentiated group (G1) and other differentiation groups (G2 and G3/G4). Anyway, it is necessary to study the biological function of genes in non-intersection set (G2 vs G3/G4). Therefore, the biological function of those genes in non-intersection set is needed in our further research. Secondly, some in vivo and in vitro experiments are essential for elucidation of the biological roles of tumor differentiation related differentially expressed genes in pancreatic adenocarcinoma in the future work.
